# A Redox-Responsive Transcription Factor Is Critical for Pathogenesis and Aerobic Growth of Listeria monocytogenes

**DOI:** 10.1128/IAI.00978-16

**Published:** 2017-04-21

**Authors:** Aaron T. Whiteley, Brittany R. Ruhland, Mauna B. Edrozo, Michelle L. Reniere

**Affiliations:** a Graduate Group in Infectious Diseases and Immunity, School of Public Health, University of California, Berkeley, Berkeley, California, USA; b Department of Microbiology, University of Washington, Seattle, Washington, USA; University of Illinois at Chicago

**Keywords:** intracellular bacteria, pathogenesis, redox signaling, virulence regulation

## Abstract

Bacterial pathogens have evolved sophisticated mechanisms to sense and adapt to redox stress in nature and within the host. However, deciphering the redox environment encountered by intracellular pathogens in the mammalian cytosol is challenging, and that environment remains poorly understood. In this study, we assessed the contributions of the two redox-responsive, Spx-family transcriptional regulators to the virulence of Listeria monocytogenes, a Gram-positive facultative intracellular pathogen. Spx-family proteins are highly conserved in Firmicutes, and the L. monocytogenes genome contains two paralogues, *spxA1* and *spxA2*. Here, we demonstrate that *spxA1*, but not *spxA2*, is required for the oxidative stress response and pathogenesis. SpxA1 function appeared to be conserved with the Bacillus subtilis homologue, and resistance to oxidative stress required the canonical CXXC redox-sensing motif. Remarkably, *spxA1* was essential for aerobic growth, demonstrating that L. monocytogenes SpxA1 likely regulates a distinct set of genes. Although the Δ*spxA1* mutant did not grow in the presence of oxygen in the laboratory, it was able to replicate in macrophages and colonize the spleens, but not the livers, of infected mice. These data suggest that the redox state of bacteria during infection differs significantly from that of bacteria growing *in vitro*. Further, the host cell cytosol may resemble an anaerobic environment, with tissue-specific variations in redox stress and oxygen concentration.

## INTRODUCTION

Bacteria adapt and respond to a wide variety of stressors. One of these is oxidative stress (also referred to here as redox stress), which is an imbalance in electrons that can damage DNA, iron-sulfur clusters, lipids, and proteins (1). Redox stress is both produced by the bacteria (endogenous) and encountered in the environment (exogenous) (2). Endogenous redox stressors, such as reactive oxygen species (ROS) generated from the incomplete reduction of oxygen, are constitutively produced during aerobic respiration (3). Therefore, bacteria have evolved diverse detoxification mechanisms to survive in oxygen-rich environments, including production of antioxidants and enzymes that consume damaging ROS. The same detoxification strategies that bacteria use to survive endogenous oxidative stress have been adopted by bacterial pathogens to thrive under the exogenous stress conditions encountered during infection (as reviewed elsewhere [1, 2, 4, 5]). Exogenous sources of redox stress are abundant within a mammalian host, most notably, during the respiratory burst generated by phagocytes and aimed at defending against invading pathogens (2). During this assault, ROS, such as superoxide anions, hydroxyl radicals, and hydrogen peroxide, as well as reactive nitrogen species (RNS), such as nitric oxide and peroxynitrite, are produced (6). To survive the hostile host environment and cause disease, bacterial pathogens have developed mechanisms to detect and adapt to the myriad redox stressors.

The Gram-positive facultative intracellular pathogen Listeria monocytogenes is an excellent model for studying redox regulation during pathogenesis, as it is able to adapt to a wide variety of conditions and has a well-characterized infectious life cycle (7). The intracellular life cycle of L. monocytogenes begins when the bacterium is phagocytosed by a host cell, where it transiently resides within the oxidizing environment of the phagosome (8). L. monocytogenes then secretes the pore-forming toxin listeriolysin O (LLO; encoded by *hly*) to escape from the phagosome and enter into the cytosol (9). The host cytosol is a highly reducing environment containing millimolar concentrations of the low-molecular-weight thiol antioxidant glutathione (10). In this reducing environment, L. monocytogenes replicates and expresses ActA, which mediates host actin polymerization and allows the bacterium to move within the cell as well as spread to neighboring cells without entering the extracellular space (11). Within 30 min, the bacteria transit from the oxidizing phagosome to the reducing cytosol (8), making L. monocytogenes an ideal model for studying adaptive responses to redox changes during pathogenesis.

To sense and adapt to redox stress, the genomes of many Firmicutes, including the genome of L. monocytogenes, encode one or more copies of an arsenate reductase (ArsC)-family protein, named Spx in the model organism Bacillus subtilis (12). Spx is a global regulator that activates and represses transcription in response to oxidative stress via direct interaction with the α subunit of RNA polymerase (RNAP) (12–15). Oxidative stress is sensed through the conserved cysteine-X-X-cysteine (CXXC) motif, which is reduced under normal growth conditions. Upon encountering oxidative stress, the cysteine residues of the Spx CXXC motif form an intramolecular disulfide bond that stabilizes the Spx-RNAP-DNA interaction and allows the Spx-mediated activation of transcription (16). In B. subtilis, over 100 genes are activated in an Spx-dependent manner, including those important for thiol homeostasis, such as thioredoxin, thioredoxin reductase, and bacillithiol biosynthesis (15, 17, 18). In addition to maintaining redox homeostasis, Spx homologues in related organisms regulate organosulfur metabolism (19), cell wall homeostasis (20, 21), competence (22), and biofilm formation (23). Spx also has anti-alpha factor activity, as it represses over 170 genes, including the biosynthetic machinery for amino acids, vitamins, and nucleic acids (15). Spx-family proteins have been demonstrated to be important for the virulence of Enterococcus faecalis (24), Streptococcus mutans (25, 26), and Streptococcus sanguinis (27).

The L. monocytogenes genome contains two *spx* orthologues: *spxA1* (*lmo2191*) and *spxA2* (*lmo2426*). SpxA1 shares 83% amino acid sequence identity with B. subtilis Spx and 25% amino acid sequence identity with SpxA2. *spxA1* is reported to be essential (28), but we recently identified a transposon insertion in a promoter of *spxA1* that results in a 10-fold reduction in *spxA1* expression (29). The *spxA1* knockdown strain is more sensitive to oxidative stress, impaired for growth, and significantly attenuated in a murine model of infection, suggesting an important role for *spxA1* and redox homeostasis in virulence (29). Here, we further examined the role of *spxA1* and *spxA2* in L. monocytogenes pathogenesis. Specifically, we demonstrated that *spxA1* is essential for aerobic growth and report that *spxA1* can be deleted only under anaerobic conditions. Surprisingly, the Δ*spxA1* mutant was capable of replicating in the host cytosol and colonizing the spleens of infected mice, although it was significantly attenuated compared to the wild-type strain. In contrast, *spxA2* was not required for virulence, demonstrating that *spxA1* is the dominant Spx-family member required for the oxidative stress response during L. monocytogenes infection.

## RESULTS

### SpxA2 is not required for the oxidative stress response or intracellular growth.

**FIG 1:**
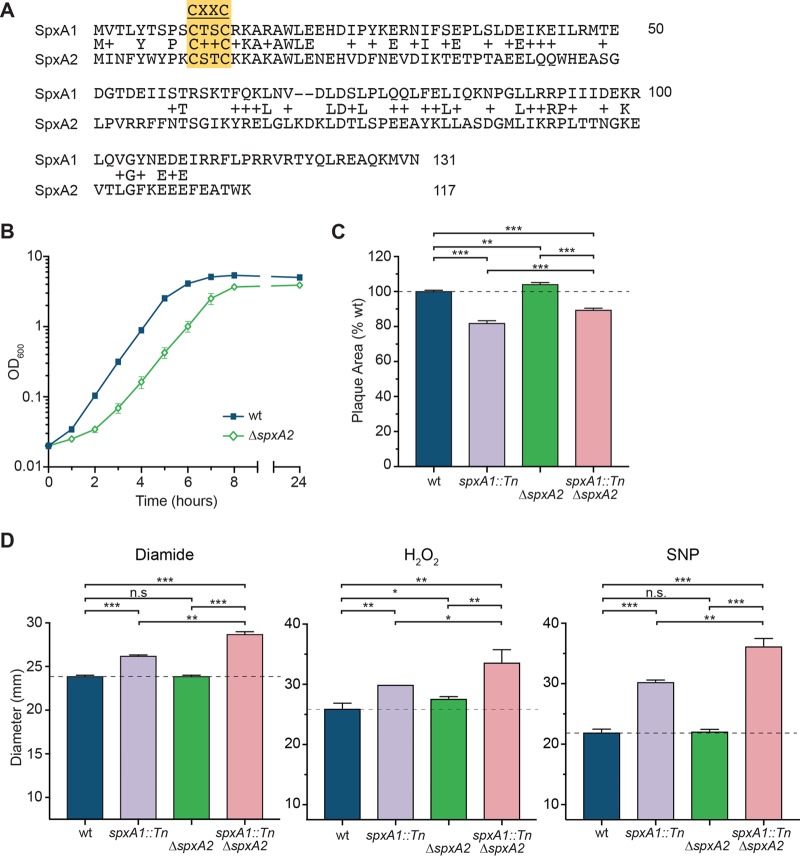
SpxA2 is not required for the disulfide stress response or cell-to-cell spread. (A) Alignment of L. monocytogenes SpxA1 and SpxA2. Yellow box, the CXXC motif. Identical residues are indicated, and amino acids of similar charge are marked with a plus sign. (B) Growth obtained in flasks with shaking at 37°C. Data are the means and SEMs from three independent experiments. (C) Plaque area measured as a percentage of that of the wt strain. Data are the means and SEMs from three independent experiments. (D) Sensitivity to disulfide stress was measured by growth inhibition in a disk diffusion assay using diamide (1 M solution), hydrogen peroxide (H_2_O_2_; 5% solution), or sodium nitroprusside (SNP; 2 M solution). The diameters of the zone of clearance, including the disks (diameter, 7.5 mm; thus, the minimum value is provided), were measured after 24 h of growth in tryptic soy agar. Data are the means and SEMs from three independent experiments. In all panels, *P* values were calculated using a heteroscedastic Student's *t* test. n.s., not significant (*P* > 0.05); *, *P* < 0.05; **, *P* < 0.01; ***, *P* < 0.001.

Previous work suggested a connection between the L. monocytogenes redox response and virulence (29). These observations prompted us to investigate the role of the Spx family of global redox-responsive transcription regulators in L. monocytogenes. Like the genomes of many other Firmicutes, the genome of L. monocytogenes contains two Spx-family paralogues, *spxA1* and *spxA2*, which share 56% similarity, 25% amino acid sequence identity, and the canonical CXXC motif that is required to sense oxidative stress (Fig. 1A) (16). *spxA1* was reported to be an essential gene (28), and accordingly, we were unable to delete *spxA1* using conventional methods. In contrast, *spxA2* could be deleted by standard allelic exchange. The Δ*spxA2* strain exhibited a slight growth defect in broth, with a doubling time of 44.2 ± 1.75 min (standard error of the mean [SEM]), whereas the doubling time for the wild type (wt) was 39.9 ± 0.32 min (Fig. 1B).

To investigate the role of *spxA2* in intracellular growth and cell-to-cell spread, we utilized a plaque assay, an *in vitro* model of infection that is correlated with L. monocytogenes virulence *in vivo*. In this assay, a monolayer of L2 murine fibroblasts is infected and immobilized in agarose containing gentamicin to kill the extracellular bacteria. At 3 days postinfection, live cells are imaged and the area of each plaque formed by L. monocytogenes is a measure of cell-to-cell spread. The Δ*spxA2* mutant formed a plaque similar in size to that formed by the wt (Fig. 1C), indicating that the Δ*spxA2* mutant is not deficient for intracellular growth or cell-to-cell spread. In some Gram-positive bacteria containing two *spx* paralogues, the Spx proteins function together to modulate the transcriptional response to oxidative stress (30). To test if SpxA1 and SpxA2 cooperate in L. monocytogenes, a double mutant in which *spxA2* was deleted and *spxA1* expression was significantly reduced via a transposon in the *spxA1* promoter was constructed (the *spxA1*::Tn Δ*spxA2* mutant) (29). The *spxA1* knockdown strain forms a plaque significantly smaller than that formed by the wt. However, the deletion of *spxA2* in this background did not reduce the ability of L. monocytogenes to grow intracellularly or spread from cell to cell (Fig. 1C). Together, these data suggest that *spxA2* is not required for pathogenesis.

In other Firmicutes, Spx-family proteins are required to survive the oxidative stress imposed by the thiol-oxidizing compound diamide (15, 23, 26). Therefore, we used a disk diffusion assay to analyze the role of *spxA2* in L. monocytogenes resistance to thiol stress. Whereas *spxA1* depletion was associated with increased sensitivity to diamide (29), the Δ*spxA2* mutant and the wt were similarly resistant (Fig. 1D), indicating that *spxA2* is not required for the L. monocytogenes response to disulfide stress. In addition to disulfide stress, *spxA1* was required for the response to peroxide and nitrosative stress, while *spxA2* had only a minor effect on the resistance to peroxide (Fig. 1D). Interestingly, the double mutant was significantly more sensitive than the single mutants to all three oxidative stressors, demonstrating that the function of SpxA2 may be revealed only in the absence of SpxA1. These data suggest that SpxA1 and SpxA2 may function cooperatively in response to a variety of redox stressors.

### SpxA1 function is conserved between L. monocytogenes and B. subtilis.

**FIG 2:**
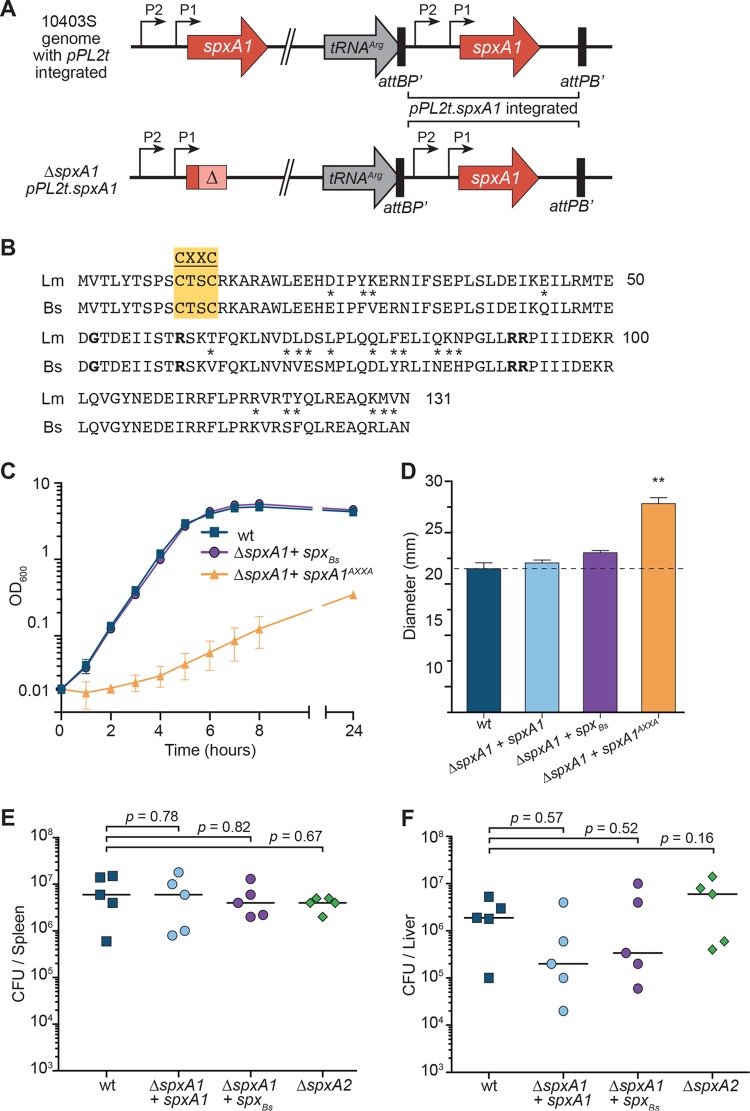
SpxA1 function is conserved with B. subtilis Spx. (A) Schematic of the *spxA1* merodiploid strain. *spxA1* is predicted to have two transcription start sites (32), pictured as thin black arrows with the labels P2 and P1. pPL2t.*spxA1* is integrated at the tRNA^Arg^ site and can be replaced with a chloramphenicol-resistant pPL2 vector expressing other alleles of *spxA1* via generalized transduction (31). (B) Alignment of L. monocytogenes SpxA1 (Lm) and B. subtilis Spx (Bs). Asterisks, residues that differ; yellow box, the CXXC motif; boldface, other functional Spx residues (G52, R60, R91, and R92) (13, 33, 34). (C) Growth obtained in flasks with shaking at 37°C. Data are the means and SEMs from three independent experiments. (D) Sensitivity to disulfide stress was measured by growth inhibition in a disk diffusion assay using a 1 M diamide solution, as described in the legend to Fig. 1D. Data are the means and SEMs from three independent experiments. *P* values were calculated using a heteroscedastic Student's *t* test. **, *P* < 0.01. (E and F) Female CD-1 mice were infected with 10^5^ CFU intravenously, and organs were harvested at 48 h postinfection. Each symbol represents an individual mouse, and the horizontal lines indicate the median. Data are for 5 mice per strain. *P* values were calculated using a heteroscedastic Student's *t* test.

SpxA1 homologues in related organisms are often not essential, and we postulated that (i) SpxA1 in L. monocytogenes has evolved additional protein functions, (ii) SpxA1 regulates a distinct set of genes, or (iii) L. monocytogenes is uniquely sensitive to redox stress. To overcome the challenges of investigating an essential gene, a strain that was merodiploid for *spxA1* was constructed (Fig. 2A). This bacterium harbored a second copy of *spxA1*, expressed from its predicted promoters at a neutral locus in the chromosome (tRNA^Arg^) via a tetracycline-resistant version of the integration vector pPL2 (31, 32). The endogenous *spxA1* gene was then deleted using standard allelic exchange techniques. Subsequently, various alleles of *spxA1* could replace the remaining wild-type copy of *spxA1* via generalized transduction using bacteriophage and a chloramphenicol-resistant pPL2 vector (Fig. 2A). This approach enabled interrogation of multiple SpxA1 alleles; however, attempts to generate an *spxA1*-deficient strain by transducing an empty chloramphenicol-resistant pPL2 vector into the Δ*spxA1* pPL2t.*spxA1* strain under conventional growth conditions were repeatedly unsuccessful.

The Spx family of proteins is defined by an N-terminal CXXC redox switch, which is required for Spx-mediated transcriptional activation in B. subtilis (16). In addition, several residues of B. subtilis Spx are required for its function and interaction with RNAP, including G52, R60, R90, and R91 (13, 33, 34). All of these residues are conserved between B. subtilis Spx and L. monocytogenes SpxA1, which share 95% similarity and 83% amino acid sequence identity overall (Fig. 2B). This high degree of similarity suggested that the molecular function(s) of Spx may be conserved, so we next tested if *spx* from B. subtilis (*spx_Bs_*) was sufficient to complement Δ*spxA1*. Using the approach described above (Fig. 2A), the Δ*spxA1* strain was complemented with B. subtilis spx under the control of the L. monocytogenes spxA1 promoters (Δ*spxA1* + *spx_Bs_*), *spxA1* in which the cysteine residues of the CXXC motif were mutated to alanine (AXXA; *spxA1*^*AXXA*^), or the wt *spxA1* allele. We obtained equivalent numbers of transductants during construction of these strains, suggesting that *spx_Bs_* and *spxA1*^*AXXA*^ were sufficient to complement the essential functions of *spxA1* and were unlikely to harbor suppressor mutations (data not shown). The strain expressing *spx_Bs_* grew like the wt in rich brain heart infusion (BHI) broth (Fig. 2C). However, the strain expressing the *spxA1*^*AXXA*^ allele grew very poorly, with a doubling time of 119.6 ± 4.8 min (Fig. 2C). Further, in the strains expressing *spxA1* and *spx_Bs_* but not the strain expressing *spxA1*^*AXXA*^, resistance to disulfide stress was restored and the strains exhibited a level of resistance to diamide similar to that of the wt (Fig. 2D). Together, these data demonstrate the importance of the SpxA1 CXXC motif for L. monocytogenes growth and the disulfide stress response.

We next assessed the complementation of Δ*spxA1* by L. monocytogenes *spxA1* or *spx_Bs_* and the role of *spxA2* during infection. Both of the Δ*spxA1* strains complemented with *spxA1* or *spx_Bs_* were capable of colonizing the spleens and livers of infected mice similarly to the wt (Fig. 2E and F). Consistent with the plaque data, the Δ*spxA2* mutant was also fully virulent *in vivo* (Fig. 2E and F), demonstrating that *spxA2* is not required for pathogenesis. Together, these data underscore the functional conservation of Spx-family proteins across the Firmicutes and demonstrate that *spxA1* may be essential due to the divergence of the SpxA1 regulon or the physiology of L. monocytogenes, rather than a novel function of the SpxA1 protein.

### SpxA1 is essential for aerobic growth.

**FIG 3:**
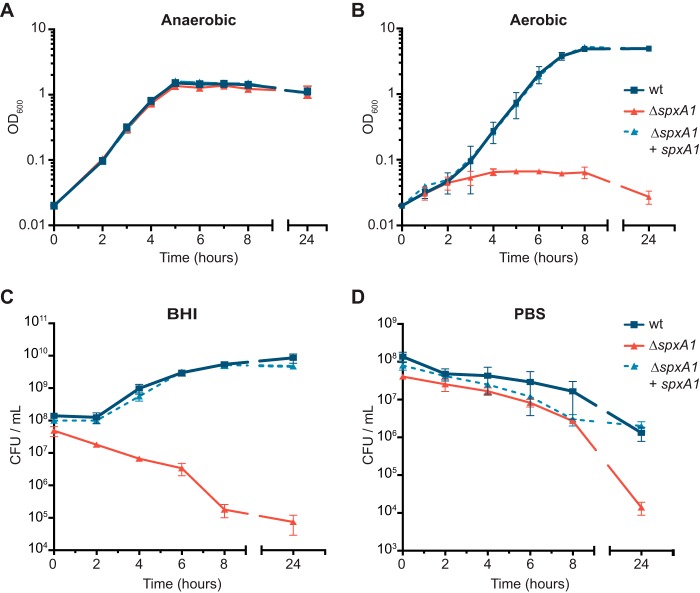
*spxA1* is essential for aerobic growth. (A) Anaerobic growth under static conditions at 37°C was measured by determination of the OD_600_. The definitions of the symbols identified in the key are the same in all panels. (B) Curve of aerobic growth obtained in flasks with shaking at 37°C. (C and D) Oxygen toxicity was measured by diluting anaerobic stationary-phase cultures into BHI or PBS and incubating aerobically at 37°C with shaking. The number of CFU per milliliter was measured over time by plating serial dilutions anaerobically on BHI. The data in all panels represent the means and SEMs from three independent experiments.

SpxA1 appeared to be critical to the redox stress response of L. monocytogenes (Fig. 1D) (29). We reasoned that this hypersensitivity to oxidative stress might render *spxA1*-deficient strains unable to cope with the endogenous redox stress generated during aerobic respiration. In support of this hypothesis, E. faecalis Δ*spx* mutants were obtained only during anaerobic growth (24). Using the approach described above (Fig. 2A), we generated *spxA1*-null mutants by transducing an empty pPL2 vector and growing the bacteria anaerobically. Transductants incubated anaerobically at 37°C appeared within the same time frame as the wt, and the colonies were similar in size (data not shown). When these Δ*spxA1* mutants were inoculated anaerobically into deoxygenated BHI broth, they grew similarly to the wt (Fig. 3A). However, after dilution into aerobic broth, the Δ*spxA1* mutant was unable to grow aerobically in a shaking flask (Fig. 3B). To investigate the kinetics of oxygen toxicity to the Δ*spxA1* mutant, anaerobic stationary-phase cultures were diluted into BHI or phosphate-buffered saline (PBS) and incubated at 37°C with shaking. Samples were removed, and serial dilutions were plated and incubated anaerobically to enumerate the surviving bacteria. In rich medium, the wt and the complemented strain grew with normal logarithmic kinetics, while the growth of the Δ*spxA1* mutant decreased ∼10-fold in 6 h and approximately 1,000-fold after 24 h (Fig. 3C). All three strains survived similarly in PBS for 8 h, while the growth of the Δ*spxA1* mutant compared to that of the wt was reduced ∼2 log units 24 h after inoculation (Fig. 3D). These data demonstrate that *spxA1* is essential for the aerobic growth of L. monocytogenes but that oxygen is not rapidly toxic in the absence of *spxA1*.

### The Δ*spxA1* mutant grows intracellularly and colonizes the spleens of infected mice.

**FIG 4:**
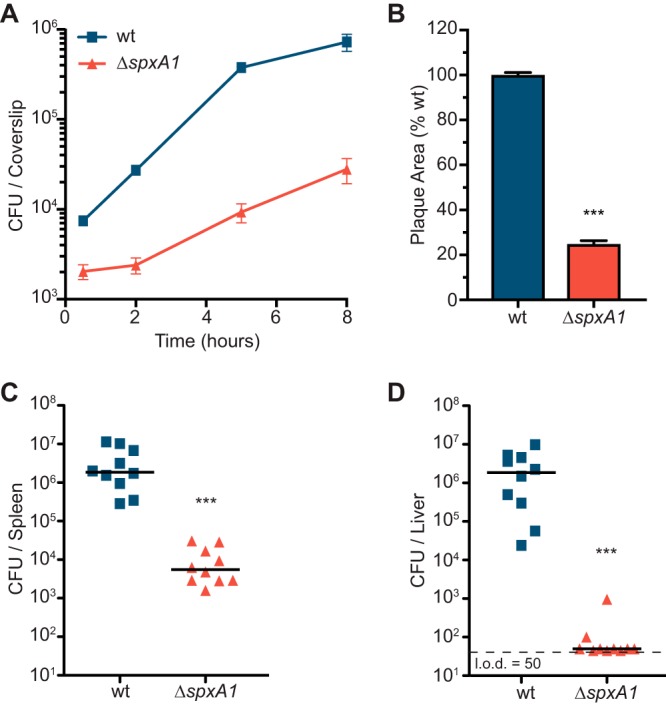
Δ*spxA1* mutants grow in macrophages and colonize mice. (A) Curves of intracellular growth of L. monocytogenes strains in BMMs. Data are the means and SEMs from three independent experiments. (B) Plaque area measured as a percentage of that for the wt strain. Data are the means and SEMs from three independent experiments. (C and D) Female CD-1 mice were infected with 10^5^ CFU intravenously, and organs were harvested at 48 h postinfection. Each symbol represents an individual mouse, and the horizontal lines are the medians. Data are for 10 mice per strain. *P* values were calculated using a heteroscedastic Student's *t* test. ***, *P* < 0.001. The limit of detection (l.o.d.) is indicated with a dashed line.

The role of SpxA1 in virulence could now be directly addressed by constructing and culturing Δ*spxA1* mutants anaerobically. Bone marrow-derived macrophages (BMMs) were infected with anaerobic cultures of the wt or the Δ*spxA1* mutant, and bacteria harvested at each time point were plated anaerobically to enumerate the CFU. Remarkably, the Δ*spxA1* strain was able to replicate in the host cytosol and grew ∼20-fold during the 8 h of infection (Fig. 4A). In a plaque assay of cell-to-cell spread, the Δ*spxA1* mutant formed plaques 25% of the size of wt plaques (Fig. 4B). We previously observed that an *spxA1* knockdown strain was deficient for vacuolar escape and that its plaque defect was restored by overexpression of *hly*, encoding the pore-forming toxin LLO (29). To test the hypothesis that the Δ*spxA1* mutant may also be impaired in vacuolar escape and therefore unable to form a plaque, LLO abundance was analyzed by immunoblotting after anaerobic growth at 30°C, the conditions under which the bacteria were incubated prior to infection. However, under these growth conditions, the Δ*spxA1* mutant did not exhibit a defect in LLO secretion (see Fig. S1A in the supplemental material). Further, the overexpression of *hly* in the Δ*spxA1* mutant did not alter the overall kinetics of intracellular growth in BMMs (Fig. S1B).

To examine the role of *spxA1* during infection of a mammalian host, bacteria were cultured anaerobically before 10^5^ CFU of the bacteria was used to infect female CD-1 mice intravenously via the tail vein. At 48 h postinfection, the spleens and livers were harvested and homogenized and the bacteria were incubated on BHI agar anaerobically. The Δ*spxA1* strain was able to colonize the spleens of infected mice, although it was attenuated over 500-fold compared to the wt (Fig. 4C). In contrast, only 2 of the 10 mice had recoverable numbers of CFU of the Δ*spxA1* mutant in their livers (Fig. 4D). Overexpression of *hly* during infection did not significantly increase the virulence of the Δ*spxA1* mutant (Fig. S1C and D), demonstrating that the attenuated virulence of this mutant is not simply due to an inability to escape the vacuole.

## DISCUSSION

In this study, we investigated the adaptation of L. monocytogenes to diverse redox environments, including those found during infection. The L. monocytogenes genome contains two Spx-family transcriptional regulators, *spxA1* and *spxA2*, that are predicted to modulate gene expression in response to redox stress. Only *spxA1* and not *spxA2* was required for the oxidative stress response, aerobic growth, and virulence. However, SpxA1 and SpxA2 may have overlapping functions during oxidative stress *in vitro*, as the double mutant (*spxA1*::Tn Δ*spxA2*) was more sensitive to a variety of stressors than either single mutant.

The role of the Δ*spxA1* mutant during infection was directly interrogated by constructing and maintaining the mutant anaerobically. The Δ*spxA1* mutant failed to replicate in nutrient broth in the presence of oxygen, but it was able to access the host cytosol and replicate intracellularly in macrophages, suggesting that the redox environment of the host cytosol may be less stressful than the growth environment in aerobic broth. These data were also consistent with the findings obtained of a mouse model of infection, in which the Δ*spxA1* mutant colonized the spleens of infected mice. However, the Δ*spxA1* mutant replicated at a reduced rate compared to the wt both in cell culture and *in vivo*, and the livers of infected mice were nearly sterilized of the Δ*spxA1* mutant, a defect that may be a result of organ-specific differences in the oxygen concentration or redox stress. Our previous study found that an *spxA1* knockdown strain is impaired in cell-to-cell spread, a phenotype that is rescued by the constitutive expression of *hly*, which encodes LLO and is absolutely required for phagosomal escape (29). These data suggest a role for *spxA1* in escape from the primary vacuole. Here, we found that a strain lacking *spxA1* produced an amount of LLO equivalent to that produced by the wt *in vitro*, and increased expression of *hly* did not improve the growth of the Δ*spxA1* mutant in macrophages or virulence in mice. Together, these data indicate that vacuolar escape is not the rate-limiting step for the intracellular growth of the Δ*spxA1* mutant *in vivo*.

Experiments demonstrating that B. subtilis Spx can functionally replace L. monocytogenes SpxA1 during infection suggest that the physical mechanism by which these Spx-family proteins interact with RNAP to regulate transcription is conserved. However, the regulons of SpxA1 and B. subtilis Spx are likely distinct because *spxA1* is essential for L. monocytogenes aerobic growth, while the B. subtilis Δ*spx* mutant does not display a growth defect (35). Spx-dependent transcriptional activation in B. subtilis requires the formation of an intramolecular disulfide bond at the N-terminal CXXC motif (16). Consequently, genes required for the response to disulfide stress (e.g., the thioredoxin gene) are activated only upon oxidation of Spx. However, Spx-dependent transcriptional repression does not require Spx oxidation (36). The function of L. monocytogenes redox sensing via the SpxA1 CXXC motif is not yet known. Our results demonstrated that the CXXC motif is not absolutely required for aerobic growth, although mutants expressing the AXXA allele were severely impaired for growth and diamide sensitivity. These data suggest that SpxA1-mediated transcriptional repression is required for aerobic growth, while SpxA1-dependent transcriptional activation is required to respond to oxidative stress. Ongoing experiments aim to clarify these findings to determine the role of SpxA1 redox sensing in L. monocytogenes pathogenesis.

The first attempt to generate an L. monocytogenes *spxA1* mutant reported that the *spxA1* gene is essential (28), and we advanced this observation by demonstrating that *spxA1* can be deleted anaerobically, similarly to *spx* in E. faecalis (24). The Staphylococcus aureus spx orthologue was also recently reported to be essential, but suppressor mutations that allow the aerobic culture of Δ*spx* mutants under normal laboratory conditions arise (37). The S. aureus suppressor mutations are located in *rpoB* and are postulated to alter the way in which the RNAP holoenzyme interacts with the promoter sequences of essential genes. We were unable to isolate suppressor mutants of the L. monocytogenes Δ*spxA1* strain and do not yet know if similar *rpoB* mutations would alleviate *spxA1* essentiality.

Several other Firmicutes are similar to L. monocytogenes, in that their genomes encode multiple Spx-family proteins that function either cooperatively or independently. For example, in Streptococcus pneumoniae, *spxA1* and *spxA2* can each be deleted independently, but the simultaneous deletion of both is lethal, suggesting that they may have overlapping regulons (22). In Bacillus anthracis, transcriptomics revealed that *spxA1* and *spxA2* are expressed at distinct phases of growth and largely regulate the same genes (38, 39). L. monocytogenes SpxA1 is clearly the dominant Spx-family protein required for virulence and aerobic growth. However, deletion of *spxA2* in the *spxA1* knockdown strain significantly increased the sensitivity of L. monocytogenes to disulfide, peroxide, and nitrosative stressors *in vitro*. These data suggest that SpxA1 and SpxA2 may cooperate in coordinating the response to oxidative stress and may regulate common genes. More research is required to determine the function of *spxA2* in L. monocytogenes physiology.

Spx homologues positively and negatively regulate over 200 genes (15, 18). In L. monocytogenes, SpxA1-dependent transcriptional changes enable aerobic growth and virulence, though it is unclear if these activities require regulation of the same genes. Ongoing studies to characterize the SpxA1 regulon aim to identify the genes that are required for growth and define why *spxA1* is not essential during infection. These analyses will allow us to differentiate whether changes in bacterial metabolism, the relative abundance of oxygen, or the extracellular abundance of reducing agents allow Δ*spxA1* mutants to replicate *in vivo*.

## MATERIALS AND METHODS

### Ethics statement.

This study was carried out in strict accordance with the recommendations in the *Public Health Service Policy on Humane Care and Use of Laboratory Animals* (40). All protocols were reviewed and approved by the Animal Care and Use Committee at the University of California, Berkeley (AUP-2016-05-8811).

### Bacterial strains and culture conditions.

L. monocytogenes mutants were derived from wild-type strain 10403S and cultured in brain heart infusion (BHI) at 37°C with shaking, unless otherwise stated. All chemicals were purchased from Sigma-Aldrich unless otherwise stated. Antibiotics were used at the following concentrations: streptomycin, 200 μg ml^−1^; chloramphenicol, 10 μg ml^−1^ (Escherichia coli) and 7.5 μg ml^−1^ (L. monocytogenes); tetracycline, 2 μg ml^−1^; carbenicillin, 100 μg ml^−1^; and erythromycin, 1 μg ml^−1^. The L. monocytogenes strains used in this study are listed in Table 1, and the E. coli strains and plasmids used in this study are listed in Table 2. Plasmids were introduced into E. coli via chemical competence and heat shock and introduced into L. monocytogenes via *trans*-conjugation from E. coli SM10 (41).

**TABLE 1:** L. monocytogenes strains used in this study

Strain	Description	Reference or source
10403S	wt	54
MLR-L470	Δ*spxA2*	This study
MLR-L232	Δ*spxA1* pPL2t.*spxA1*	This study
MLR-L472	Δ*spxA1* pPL2.*spxA1*	This study
MLR-L637	Δ*spxA1* pPL2^*a*^	This study
MLR-L473	Δ*spxA1* pPL2.*spx_Bs_*	This study
MLR-L613	Δ*spxA1* pPL2.*spxA1^AXXA^*	This study
MLR-L609	wt pH-*hly*	49
MLR-L614	Δ*spxA1* pH-*hly*	This study

a Referred to here as the Δ*spxA1* mutant.

**TABLE 2:** Plasmids and E. coli strains used in this study

Strain	Description	Reference or source
XL1	For vector construction	Stratagene
SM10	For *trans*-conjugation	41
MLR-E011	pKSV7-oriT	42
MLR-E006	pPL2	31
MLR-E234	pPL2t	43
MLR-E480	SM10/pKSV7Δ*spxA2*	This study
MLR-E184	SM10/pKSV7Δ*spxA1*	This study
MLR-E228	SM10/pPL2t.*spxA1*	This study
MLR-E481	SM10/pPL2.*spxA1*	This study
MLR-E482	SM10/pPL2.*spx_Bs_*	This study
MLR-E607	SM10/pPL2.*spxA1^AXXA^*	This study
MLR-E530	SM10/pH-*hly*	49

### Vector construction and cloning.

The oligonucleotides used in this study are listed in Table S1 in the supplemental material. To delete *spxA2*, pKSV7Δ*spxA2* was constructed by amplifying a 5′ homologous region with primers ΔspxA2-H1-f/r and a 3′ homologous region with primers ΔspxA2-H2-f/r. Synthesis by overlapping extension (SOE) PCR was used to join the fragments together. This cassette was restriction digested and ligated into pKSV7-oriT (42). An analogous protocol was performed to delete *spxA1*, using primers ΔspxA1-H1-f/r and ΔspxA1-H2-f/r.

The *spxA1* complementation vector pPL2.*spxA1* was constructed by amplifying *spxA1* and its endogenous promoters with primers spxA1-f/r. This fragment was digested and ligated into pPL2 (chloramphenicol resistant) or pPL2t (tetracycline resistant) (31, 43). The B. subtilis *spx* complementation vector pPL2.*spx_Bs_* was constructed by amplifying *spx* from B. subtilis (strain 168) with primers Bs-spx-f/r and the L. monocytogenes spxA1 promoter region with primers P-spxA1-f/r. SOE PCR joined the two products together, and this construct was digested and ligated into pPL2 (31). pPL2.*spxA1* C10AC13A (*spxA1*^*AXXA*^) was constructed by site-directed mutagenesis (inverse PCR) of pPL2.*spxA1* with primers CXXC-f/r. The PCR product was treated with DpnI and transformed into E. coli. The sequences of all plasmids were confirmed by Sanger DNA sequencing. The nomenclature used for genetic loci is that for the EGD-e strain of L. monocytogenes by convention. However, all genetic material used here was derived from 10403S strains of L. monocytogenes, and the homologous loci are listed as follows: *spxA1*, *lmo2191*, *LMRG_01641* and *spxA2*, *lmo2426*, *LMRG_01822*.

### L. monocytogenes strain construction.

In-frame deletions were carried out by allelic exchange using a conjugation-proficient version of the suicide vector pKSV7 (42). Vectors bearing the mutant Δ*spxA1* and Δ*spxA2* alleles were introduced into L. monocytogenes via *trans*-conjugation and integrated into the chromosome, and colonies were purified on selective nutrient agar and subsequently cured of the plasmid by conventional methods (29). Chromosomal mutations were confirmed by PCR and Sanger DNA sequencing when necessary. The knock-in of genes into L. monocytogenes was carried out using the pPL2 and pPL2t integration plasmids (31, 43). Integration was confirmed by antibiotic resistance.

### Disk diffusion assays.

The zone of inhibition was measured as previously described (29, 44, 45). Briefly, L. monocytogenes was grown overnight in tryptic soy broth (TSB) at 37°C with shaking. The stationary-phase cultures (∼6 × 10^7^ CFU) were added to molten top agar (0.8% NaCl, 0.8% Bacto agar) and spread evenly over tryptic soy agar (TSA) plates. After the agar solidified, Whatman paper disks soaked in 10 μl of diamide (a 1 M solution), hydrogen peroxide (a 5% [vol/vol] solution), or sodium nitroprusside (a 2 M solution) were placed on the immobilized lawn of bacteria, and the plates were incubated at 37°C for 24 h. The diameter of the zone of inhibition was then measured with a ruler.

### Plaque assays.

Plaque assays were carried out by conventional methods (46, 55). In brief, 6-well tissue culture-treated dishes were seeded with 1.2 × 10^6^ L2 murine fibroblasts per well. The L. monocytogenes strains were incubated overnight at 30°C in BHI in a stationary culture. Overnight cultures were diluted 1:10 in sterile PBS, and 5 μl was used to infect each well of L2 cells. At 1 h postinfection, the cells were washed twice with PBS, followed by addition of 3 ml of molten agarose-Dulbecco modified Eagle medium (DMEM) solution. This solution consisted of gentamicin at 10 μg ml^−1^ and a 1:1 mixture of 2× DMEM (Gibco) and 1.4% SuperPure agarose LE (U.S. Biotech Sources, LLC). At 3 days postinfection, 2 ml of molten agarose-DMEM solution containing neutral red (Sigma) was added to each well to visualize the plaques. After 24 h, the plaques were scanned and the area was measured using ImageJ software (47).

### Growth curves.

For anaerobic growth, L. monocytogenes colonies were inoculated into broth and incubated in closed containers containing anaerobic gas-generating pouches (GasPak EZ; BD). For curves of the growth in broth, the optical density at 600 nm (OD_600_) of the overnight cultures was measured, and the cultures were normalized to an optical density of 0.02 in either 25 ml BHI in 250-ml flasks (for aerobic growth) incubated with shaking (220 rpm) at 37°C or 10 ml BHI in culture tubes (for anaerobic growth) placed in an anaerobic chamber and incubated statically at 37°C. The OD_600_ was measured every hour.

Intracellular growth curves were performed as previously described (48, 49). Briefly, bone marrow-derived macrophages (BMMs) were harvested as previously reported (50) and seeded at a concentration of 3 × 10^6^ cells in 5 ml of medium in a 60-mm dish containing sterilized tissue culture-treated coverslips. Bacteria were incubated in a stationary manner at 30°C, washed, and used to infect BMMs at a multiplicity of infection (MOI) of one bacterium for every 10 cells. At 30 min postinfection, the cells were washed and medium containing gentamicin (50 μg ml^−1^) was added. At each time point, three coverslips were removed, BMMs were lysed in water, and dilutions were plated on BHI agar to enumerate the CFU.

### Virulence assays.

Infections were performed as previously described (51, 52), with the following modifications. Aerobic strains were incubated at 37°C with shaking (220 rpm), while anaerobic strains were incubated at 37°C in closed containers containing anaerobic gas-generating pouches. All strains were diluted in PBS to a concentration of 5 × 10^5^ CFU ml^−1^, and 200 μl was injected into the tail vein of 6- to 8-week-old female CD-1 mice (Charles River Laboratories). The inocula were plated after infection and incubated anaerobically to ensure consistent doses across strains. The Δ*spxA1* mutant inoculum was within ±3-fold of the wt inoculum. At 48 h postinfection, the mice were euthanized and the livers and spleens were harvested. Organs were homogenized in 0.1% (vol/vol) NP-40 in water, and serial dilutions were made in PBS and plated on BHI agar to enumerate the CFU. Plates with tissue samples from Δ*spxA1* mutant-infected mice were incubated anaerobically.

### Generalized transduction.

The U153 phage was utilized for generalized transduction as previously described (53). Briefly, transducing lysates from donor strains were constructed by mixing donors with phage at an MOI of approximately 1, incubated overnight at 30°C in LB soft agar, filter sterilized, and mixed with recipient L. monocytogenes at an MOI of 0.1 for 30 min, and transductants were selected on antibiotic-containing BHI agar at 37°C.

## Supplementary Material

Supplemental material
