# Supplementary material for: A Redox-Responsive Transcription Factor Is Critical for Pathogenesis and Aerobic Growth of Listeria monocytogenes
Source: Infect Immun. 2017 Apr 21;85(5):e00978-16. doi: 10.1128/IAI.00978-16 (PMC5400837; doi:10.1128/IAI.00978-16)
Supplement: Supplemental material [file supp_85_5_e00978-16__index.html]

Supplemental material 

# A Redox-Responsive Transcription Factor Is Critical for Pathogenesis and Aerobic Growth of Listeria monocytogenes

## Supplemental material

- Supplemental file 1 -

  Supplemental methods. Fig. S1. Overexpression of LLO is not sufficient to rescue ∆*spxA1* pathogenesis. Table S1. Oligonucleotides used in this study.

  PDF, 141K
