# Supplementary material for: A Redox-Responsive Transcription Factor Is Critical for Pathogenesis and Aerobic Growth of Listeria monocytogenes
Source: Infect Immun. 2017 Apr 21;85(5):e00978-16. doi: 10.1128/IAI.00978-16 (PMC5400837; doi:10.1128/IAI.00978-16)
Supplement: Supplemental material [file IAI.00978-16_zii999092019s1.pdf]

SUPPLEMENTAL MATERIALS

Supplemental Methods

Immunoblots

Supernatants were collected from cultures by centrifugation. Proteins were precipitated on ice with 10% v/v trichloroacetic acid for one hour, followed by three acetone washes and vacuum drying. Proteins were then dissolved in LDS buffer (Invitrogen) containing 5% v/v 2-mercaptoethanol (Sigma), in a volume normalized to the OD_{600} of the bacterial culture. Samples were then separated by SDS-PAGE and analyzed with antibodies specific for LLO or P60 (Adipogen), as previously described (1, 2).

Supplemental Figure Legend

Figure S1. Over-expression of LLO is not sufficient to rescue Δ*spxA1* pathogenesis.

A. Representative immunoblot of culture supernatants from bacteria grown to stationary phase anaerobically at 30 °C. *pH-hly* strains constitutively over-express *hly*, encoding LLO. LLO abundance was normalized to P60 abundance and presented as a percentage of wt. The mean normalized LLO abundance of four independent experiments is displayed for each sample. **B.** Intracellular growth curve of *L. monocytogenes* strains in BMMs. Data are the mean and s.e.m. of three independent experiments. **C and D.** Female CD-1 mice were infected with 10^5 CFU intravenously and organs were harvested 48 hours post-infection. Data from Figures 4C and 4D are

reproduced here to demonstrate the comparison between the wt and the *pH_hly* backgrounds. Data represent individual mice and the horizontal lines are the medians. n = 5 or 10 mice per strain. *p* values were calculated using a heteroscedastic Student's *t*-test; *** $p < 0.001$. The limit of detection (l.o.d.) is indicated with a dashed line.

Supplementary Table 1. Oligonucleotides used in this study

Primer Name	Sequence (5' - 3')[a]	Description
spxA1-3'-f	GGCC**GGATCC**TAAATGTTCTTGAAATTTTTTCGC	3' Genomic Region
spxA1-3'-r	CCGG**CTGCAG**CTCCAATGTTATTTCTGGCTG	3' Genomic Region
ΔspxA2-H1-f	GGCC**GGTACC**GCTGCAACCCGATTTAGGG	*spxA2* 3' Homologous Region
ΔspxA2-H1-r	CTATATTGACTATTTCCAAGTTGCATTAATCATTTTGCCTCTC CCT	
ΔspxA2-H2-f	AGGGAGAGGCAAAATGATTAATGCAACTTGGAAATAGTCAA TATAG	*spxA2* 5' Homologous Region
ΔspxA2-H2-r	CCGG**CTGCAG**GCCTCCGATATTTTATGTTTCAC	
ΔspxA1-H1-f	CCGG**GAGCTC**GGTTGTGGTAAATCTACAACTG	*spxA1* 3' Homologous Region
ΔspxA1-H1-r	GGTGGAATCTTATCTTAGCTGCGAAATTCTAGCAATATCTC TAT	
ΔspxA1-H2-f	ATAGAGATATTGCTAGAATTTCGCAGCTAAGATAAGATTCC ACC	*spxA1* 5' Homologous Region
ΔspxA1-H2-r	CCGG**CTGCAG**CTTCATTGTCTTCTCGTGGGC	
spxA1-f	GGCC**GGTACC**GAAAACATCAATCAGAGTTAAATT	Amp *spxA1* with native promoters for pPL2 complement
spxA1-r	GGCC**GGATCC**TTAGTTAACCATTTTTTGCGCTTCA	
Bs-spx-f	AAGGAGTGTGAATGTCTAATGGTTACACTATACACATCAC	*B. subtilis spx*
Bs-spx-r	GGCC**GGATCC**TTAGTTTGCCAAACGCTGTGCTT	
P-spxA1-f	GGCC**GGTACC**GAAAACATCAATCAGAGTTAAATT	*Lm spxA1* promoter region
P-spxA1-r	GTGATGTGTATAGTGTAACCATTAGACATTCACACTCCTT	
CXXC-f	CACTTCACCTAGTGCTACATCTGCTCGAAAAGCTCGTGC	iPCR to mutate C10 and C13 to Ala
CXXC-r	GCACGAGCTTTTCGAGCAGATGTAGCACTAGGTGAAGTG	

[a] Bold font indicates restriction enzyme site.

Supplemental References

1. **Reniere ML**, **Whiteley AT**, **Hamilton KL**, **John SM**, **Lauer P**, **Brennan RG**, **Portnoy DA**. 2015. Glutathione activates virulence gene expression of an intracellular pathogen. Nature **517**:170–173.

2. **Melton-Witt JA**, **McKay SL**, **Portnoy DA**. 2012. Development of a single-gene, signature-tag-based approach in combination with alanine mutagenesis to identify listeriolysin O residues critical for the in vivo survival of Listeria monocytogenes. Infect Immun **80**:2221–2230.

Figure S1

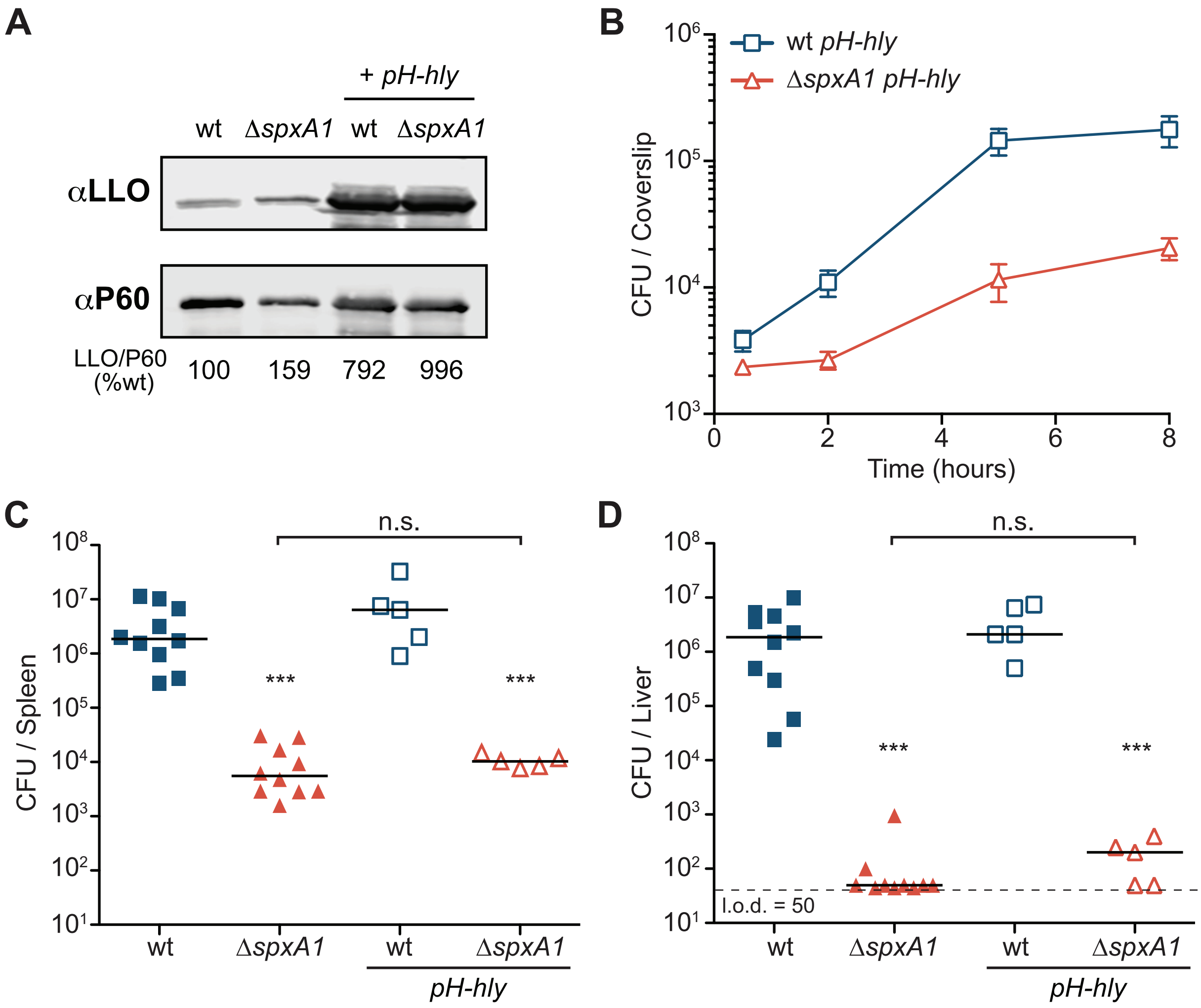
A
+ pH-hly
wt ΔspxA1 wt ΔspxA1
αLLO
αP60
LLO/P60 (%wt) 100 159 792 996
B
wt pH-hly
ΔspxA1 pH-hly
CFU / Coverslip
Time (hours)
C
n.s.
***
***
CFU / Spleen
wt ΔspxA1 wt ΔspxA1
pH-hly
D
n.s.
***
***
CFU / Liver
l.o.d. = 50
wt ΔspxA1 wt ΔspxA1
pH-hly
